# Research on the Accessibility of Different Colour Schemes for Web Resources for People with Colour Blindness

**DOI:** 10.3390/jimaging11080268

**Published:** 2025-08-11

**Authors:** Daiva Sajek, Olena Korotenko, Tetiana Kyrychok

**Affiliations:** 1 Kauno Kolegija Higher Education Institution, LT-50468 Kaunas, Lithuania; daiva.sajek@go.kauko.lt; 2 Educational and Scientific Institute for Publishing and Printing, National Technical University of Ukraine “Igor Sikorsky Kyiv Polytechnic Institute”, 03056 Kyiv, Ukraine; t_kyrychok@ukr.net

**Keywords:** colour blindness, colour schemes, accessibility, website design, user surveys

## Abstract

This study is devoted to the analysis of the perception of colour schemes of web resources by users with different types of colour blindness (colour vision deficiency). The purpose of this study is to develop recommendations for choosing the optimal colour scheme for web resource design that will ensure the comfortable perception of content for the broadest possible audience, including users with colour vision deficiency of various types (deuteranopia and deuteranomaly, protanopia and protanomaly, tritanopia, and tritanomaly). This article presents the results of a survey of people with different colour vision deficiencies regarding the accessibility of web resources created using different colour schemes. The colour deviation value ∆E was calculated to objectively assess changes in the perception of different colour groups by people with colour vision impairments. The conclusions of this study emphasise the importance of taking into account the needs of users with colour vision impairments when developing web resources. Specific recommendations for choosing the best colour schemes for websites are also offered, which will help increase the accessibility and effectiveness of web content for users with different types of colour blindness.

## 1. Introduction

Today, digital content has a significant impact on various aspects of society, contributing to its development and progress in various fields. According to data from [1], today, there are more than 200 million active websites on the Internet that are used by more than 5.16 billion users [2]. That means that 64.4% of the world’s population has access to the Internet and actively uses it. However, at least 2.2 billion people worldwide have visual impairments of various types and degrees [3].

Among vision defects, a significant share is occupied by colour blindness or colour vision deficiency (CVD)—a reduced ability to perceive colour or distinguish between colours. According to [4], more than 300 million people have colour blindness. The severity of colour blindness ranges from almost imperceptible to complete absence of colour perception, with a noticeable gender impact—approximately 1 in 12 men (8%) and 1 in 200 women (0.5%) suffer from colour blindness [5]. These data show that a large proportion of Internet users may have various barriers to accessing information and other features of web products.

The main types of colour blindness are the following: deuteranopia and deuteranomaly, which are characterised by a complete inability of the eye or disturbances in the perception of green colour (affecting approximately 1.1% of the male population and 0.02% of the female population); protanopia and protanomaly, which are characterised by the absence of or severe impairment in the perception of red colour (affecting approximately 1.0% of the male population and 0.1% of the female population); and tritanopia and tritanomaly, which cause a complete inability or disturbance in the perception of blue colour (relatively rare) [5,6,7].

One of the stages of developing a web resource is choosing a colour scheme for it. Typical colour schemes based on colour theory are often used. The most common and widespread are analogous, monochromatic, triadic, and complementary schemes [8,9]. When choosing a colour scheme for a website, it is important to consider colour associations, their psychological impact, and their interaction with content [9], as well as their accessibility to most users, including people with visual impairment or colour blindness.

The authors of [10], who are devoted to choosing a more effective colour scheme for assisting readers in locating information in a colour-coded article, bring attention to the importance of using colour coding of information. The authors emphasise that using a correctly selected colour scheme provides a significant advantage in human cognitive activity, especially in reading and understanding text information. However, this study excluded participants with visual impairment or various types of colour blindness, which discriminates against such individuals, and the results of the study can only be used for people with normal vision. The accessibility of colour schemes usually refers to the ability of people with disabilities to recognise all elements of a web resource interface, perceive them correctly, and have access to and use them [11].

Interface designers use different approaches to solve the problem of content accessibility for people with colour blindness, guided by different recommendations and research results. In particular, a large number of designers recommend using the high-contrast mode approved by the WCAG recommendations [12,13], which defines the minimum colour contrast ratio for legible text. The WCAG approves appropriate contrast ratios; in particular, it recommends that the contrast between text and background should be at least 4.5:1 for standard text and 3:1 for large text [13]. The WCAG recommendations are used quite effectively and have become the basis for various colour generators and colour palettes. However, they often contradict the aesthetic preferences of some web designers or do not match the semantic content of web products [4,14]. This necessitates additional analysis and a search for alternative solutions to the selection of colour schemes for web resources. In [4], a study was conducted on how users with CVD can evaluate the functionality and aesthetics of existing interfaces. The researchers’ attention was focused on a comparative study of the perception of interfaces by people with and without colour vision impairment. The researchers emphasise that the increased contrast of web resources is often aesthetically unappealing to users without visual impairments. Therefore, they proposed a new experimental protocol based on simulation, where users evaluated the relative aesthetics and functionality of screenshots of 20 popular webpages in full colour compared with the same pages simulated with respect to CVD. The results of their study showed a positive correlation between the relative aesthetics and functionality of electronic publications for both groups of users. However, the authors themselves emphasise that their results apply only to simulated screenshots and cannot yet be applied to all colour schemes of web resources. This creates a need for a more comprehensive approach to providing a wider choice of colour schemes for web resources, which would improve their accessibility for a wide range of users, including people with colour blindness.

The authors of [15] investigated the problem of creating accessible web content for users with low vision and colour blindness, and based on this, a model for designing and evaluating web content with accessible colour and contrast for visually impaired people was developed. This model was built as part of the process of updating the webpages of the University of Plovdiv. Therefore, only the specific colour schemes inherent in these webpages were studied. The main attention was also focused on achieving the appropriate level of contrast (again due to WCAG recommendations) to ensure the accessibility of the web resource for people with colour vision impairment. However, as noted above, this is not always a feasible method, as it may conflict with the aesthetic needs of people without visual impairments, or following the guidelines may complicate the work of web designers or web developers.

The authors of [16] discuss the problem of colour combination and the possibility of using alternative visual elements to display map data for unambiguous interpretation of information by people without and with visual impairments. The techniques presented here are intended to help cartographers create maps that can be easily read by all users, including people with colour blindness. The study describes a highly relevant problem, but the work is more about developing maps, which entails analysing specific colour palettes rather than the general colour spectrum in terms of their perception by people with colour blindness. The authors of [17] provide recommendations for improving the accessibility of websites for adult users with specific physical and cognitive limitations when interacting with a computer or accessing a website. They also recommend not using colour as the only way to indicate a visual element or to convey information, indicate an action, request a response, or make a choice. However, as with the recommendations given in [16], it is worth noting that adding additional elements (such as various labels, hatching of images, etc.) can sometimes overload and complicate the design, in addition to being aesthetically unappealing for web designers and users.

Thus, the analysis of the sources demonstrated that one of the key approaches to ensuring the accessibility of web resources for people with colour blindness is to use a high-contrast mode or introduce additional identifying elements. However, such solutions may not meet the aesthetic requirements of both users and web designers or the semantic content of web resources. The findings of the researchers confirm the need for research to ensure a balance between functionality and aesthetics of design for all user groups, including people with colour blindness. Thus, we propose a study aimed at determining the optimal colour scheme at the design stage of web resources, taking the principles of accessibility into account. The purpose of this study is to develop recommendations for choosing colour schemes that will ensure a comfortable perception of content for the broadest possible audience, including users with colour perception disorders of various types, which is extremely important in the context of the current development of information technology and society as a whole. To achieve this goal, the following research objectives were set: (1) to analyse the distortion of different colours in the perception of people with different types of colour blindness; (2) to study the perception of different types of colour schemes by people with different types of colour blindness and to determine the most optimal types of colour schemes for this group of people. The primary focus is on choosing colour combinations that provide sufficient contrast and reduce the risk of colour mixing in people with different types of colour blindness. This study used methods of modelling colour perception using specialised programs, as well as a survey of users with different types of colour blindness.

## 2. Materials and Methods

### 2.1. Analysis of the Distortion of Different Colours in the Perception of People with Different Types of Colour Blindness

The analysis of colour distortion by people with different types of colour blindness was based on the results of the analysis of simulated colour data. To increase the objectivity of the results, colour simulation was performed using various software, including Colour Oracle [18], Sim Daltonism [19], and Coblis Colour Blindness Simulator [20], which allowed us to evaluate the perception of primary colours by people with different forms of colour blindness: deuteranopia and deuteranomaly, protanopia and protanomaly, and tritanopia and tritanomaly.

The algorithm of the simulators used is based on the conversion of colours of the input image from RGB to LMS colour space [16,21,22,23] using (1), which describes the perception of colours by the human eye through three types of retinal cones (*L*—sensitive to long waves (red spectrum), *M*—sensitive to medium waves (green spectrum), and *S*—sensitive to short waves (blue spectrum)):

(1) $$\left[\begin{array}{c} L\\ M\\ S \end{array}\right]=\left[\begin{array}{ccc} 17.8824 & 43.5161 & 4.1194\\ 3.4557 & 27.1554 & 3.8671\\ 0.0300 & 0.1843 & 1.4671 \end{array}\right]\cdot \left[\begin{array}{c} R\\ G\\ B \end{array}\right]$$

They then simulate a colour vision deficit that depends on the type of colour blindness. For deuteranopia, the *M* value is reset to zero; for protanopia, the *L* value is reset; and for tritanopia, the *S* value is reset. The new values are calculated based on transformation matrices with component adaptation. To simulate anomalies (e.g., deuteranomaly or protanomaly), the cone values are not reset to zero but are attenuated by a weighting factor.

After modelling the colour defect, the *LMS* values are converted back to the *RGB* space (2) [22,23] and scaled to the range [0, 255] (3):

(2) $$\left[\begin{array}{c} R_{n}\\ G_{n}\\ B_{n} \end{array}\right]=\left[\begin{array}{ccc} 0.0809 & -0.1305 & 0.1167\\ -0.0102 & 0.0540 & -0.1136\\ -0.0004 & -0.0041 & 0.6935 \end{array}\right]\cdot \left[\begin{array}{c} L\prime \\ M\prime \\ S\prime \end{array}\right]$$

(3) $$R=\min\left(\max\left(R_{n}\times 255,0\right),255\right),\;G=\min\left(\max\left(G_{n}\times 255,0\right),255\right)\;B=min(\max\left(B_{n}\times 255,0\right),255),$$

We simulated 128 key mnemonic colours used in HTML5 [24]. All of the colours under study were grouped into 11 shade groups: neutral light, neutral dark, orange, brown, pink, purple, blue, green, cyan (blue-green), yellow, and red.

The next step was the calculation of colour deviation for the objective assessment of the deviation in the perception of different colour groups by people with colour vision deficiencies.

To do this, we converted the colour from RGB to the CIELab colour model, which allows us to model human colour perception better. The use of the CIELab colour system in this case is justified by the fact that two chromatic parameters—*a* and *b*—reflect the ratio of the green and red components of the colour, as well as blue and yellow, respectively [25]. This allows us to clearly emphasise the peculiarities of colour perception by people with different types of colour blindness.

First, we normalised the colour components *R*, *G*, *B* (4) and performed their gamma correction (5) [26]:

(4) $$R_{norm}=\frac{R}{255},\;G_{norm}=\frac{G}{255},\;B_{norm}=\frac{B}{255},$$

(5) $$\mathrm{if}\;R_{norm}\leq 0.04045,\;\mathrm{then}\;R_{lin}=\frac{R_{norm}}{12.92}\;\mathrm{else}\;R_{lin}={\left(\frac{R_{norm}+0.055}{1.055}\right)}^{2.4},\;\mathrm{if}\;G_{norm}\leq 0.04045,\;\mathrm{then}\;G_{lin}=\frac{G_{norm}}{12.92}\;\mathrm{else}\;G_{lin}={\left(\frac{G_{norm}+0.055}{1.055}\right)}^{2.4},\;\mathrm{if}\;B_{norm}\leq 0.04045,\;\mathrm{then}\;B_{lin}=\frac{R_{norm}}{12.92}\;\mathrm{else}\;B_{lin}={\left(\frac{B_{norm}+0.055}{1.055}\right)}^{2.4}$$

The linear representation is used to calculate the coordinates in the XYZ space (6), which allows us to obtain the *X*, *Y*, and *Z* coordinates as follows [27]:

(6) $$X=0.4124\cdot R_{lin}+0.3576\cdot G_{lin}+0.1805\cdot B_{lin},\;Y=0.2126\cdot R_{lin}+0.7152\cdot G_{lin}+0.0722\cdot B_{lin},\;Z=0.0193\cdot R_{lin}+0.1192\cdot G_{lin}+0.9505\cdot B_{lin}$$

To account for lighting adaptation, XYZ normalisation was performed relative to the standard white point for the D65 (7):

(7) $$X_{norm}=\frac{X}{X_{white}},Y_{norm}=\frac{Y}{Y_{white}},Z_{norm}=\frac{Z}{Z_{white}},\;X_{white}=0.95047,Y_{white}=1.0000,Z_{white}=1.08883$$

A nonlinear transformation function was used to model luminance perception:

(8) $$f\left(t\right)=\left\{\begin{array}{c} t^{1/3},\;\;\;\;\;\;\;\;\;\;\;\;\;\;\;\;\;\;\;\;\;\;\;if\;t>0.008856\;\\ 7.787\cdot t+\frac{16}{116},\;\;if\;t\leq 0.008856\; \end{array}\right\}.$$

After applying $f\left(t\right)$, the values of *LAB* = (L,a,b) were calculated as follows:

(9) $$L=116\cdot f\left(Y_{norm}\right)-16,\;a=500\cdot (f\left(X_{norm}\right)-f\left(Y_{norm}\right)),\;b=200\cdot (f\left(Y_{norm}\right)-f\left(Z_{norm}\right))$$

Colour conversion to the Lab colour system allows for the determination of the level of distortion in the perception of each colour by individuals with different types of colour blindness compared with normal vision by calculating the colour difference (10) and its component difference (11) [25]:

(10) $${∆E}_{ijk}=\sqrt{{\left(L_{ijk\;}^{\prime }-L_{ij\;}^{0}\right)}^{2}+{\left(a_{ijk\;}^{\prime }-a_{ij\;}^{0}\right)}^{2}+{\left(b_{ijk\;}^{\prime }-b_{ij\;}^{0}\right)}^{2}},$$

(11) $${{∆L}_{ijk}=L}_{ijk\;}^{\prime }-L_{ij\;}^{0},\;{{∆a}_{ijk}=a}_{ijk\;}^{\prime }-a_{ij\;}^{0},{{∆b}_{ijk}=b}_{ijk\;}^{\prime }-b_{ij\;}^{0}$$

where *j* is the element of each *i*-th colour group; $L_{ij}^{0}$, $a_{ij}^{0}$, and $b_{ij}^{0}$ are the coordinates of the colour perceived by people with normal vision; and $L_{ijk}^{\prime }$, $a_{ijk}^{\prime }$, and $b_{ijk}^{\prime }$ are the coordinates of the simulated colour according to the *k*-type of colour blindness.

In order to estimate which groups of colours are perceived with the most significant distortions by people with colour blindness and which colour component is most distorted for them, the values of ${∆E}_{ijk}$, ${∆L}_{ijk}$, ${∆a}_{ijk}$, and ${∆b}_{ijk}$ were averaged within each group.

### 2.2. Development of Visual Materials for Research on Colour Vision Deficiency

The main prototype for this study was the main page of the Helsi website. This Ukrainian healthcare management platform connects patients, doctors, and medical institutions in a single digital environment. The primary purpose of the website is to make an appointment with a doctor, manage medical information, and provide remote medical services. Given the social importance of this website, it should be made as accessible as possible to all users, including the visually impaired.

**Figure 1:**
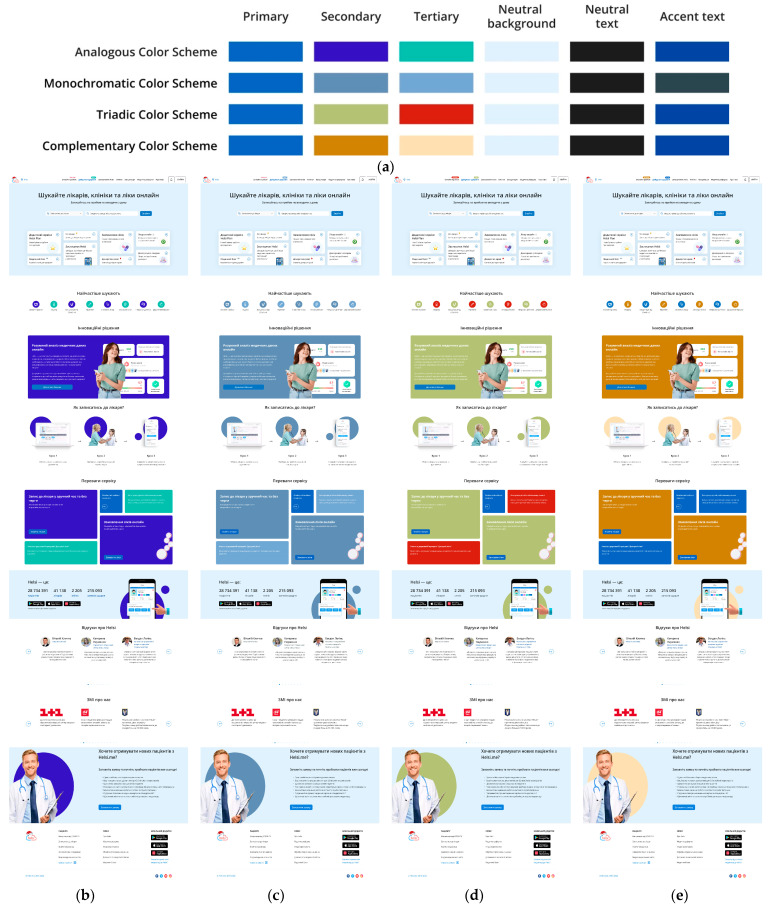
Colour schemes (analogous, monochromatic, triadic, and complementary) with blue as the primary colour (**a**) and webpage layouts built according to these schemes: analogous (**b**), monochromatic (**c**), triadic (**d**), and complementary (**e**).

**Figure 2:**
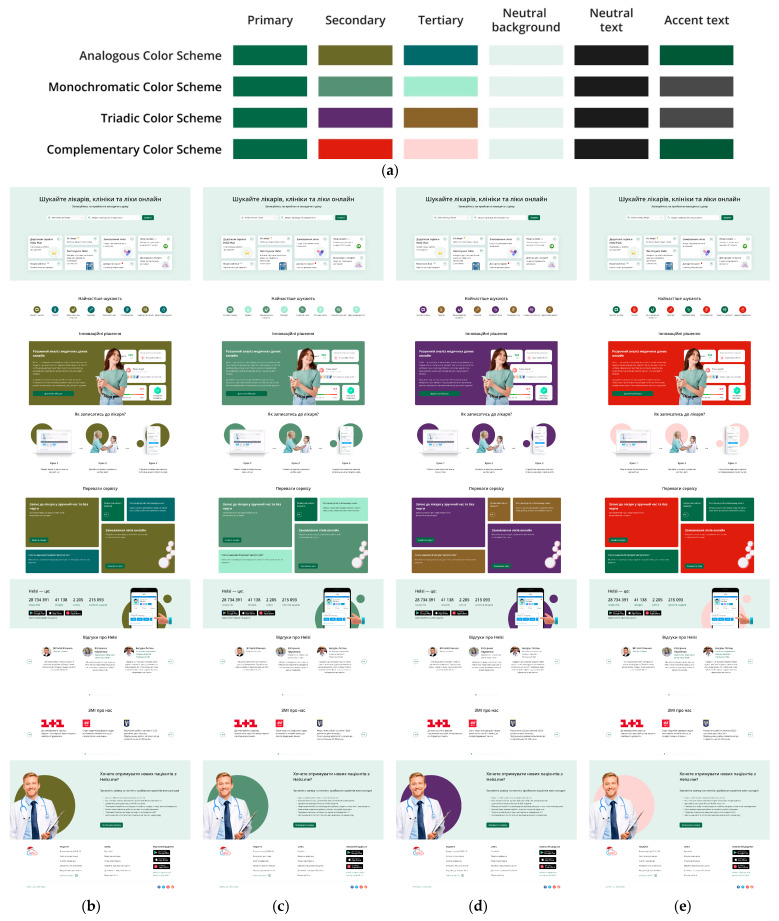
Colour schemes (analogous, monochromatic, triadic, and complementary) with green as the primary colour (**a**) and webpage layouts built according to these schemes: analogous (**b**), monochromatic (**c**), triadic (**d**), and complementary (**e**).

**Figure 3:**
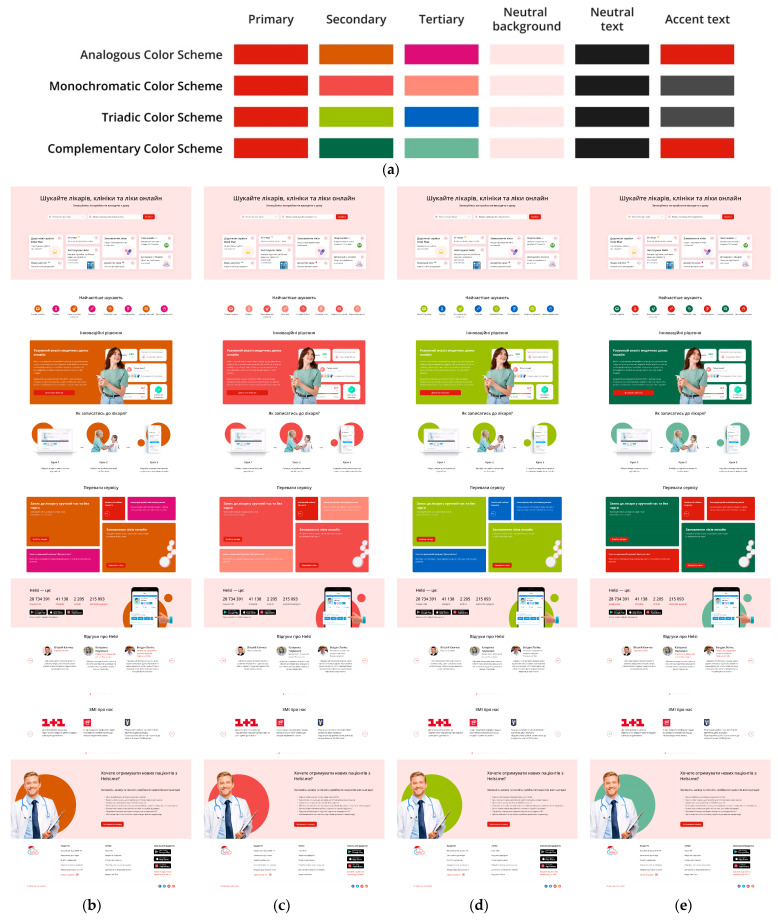
Colour schemes (analogous, monochromatic, triadic, and complementary) with red as the primary colour (**a**) and webpage layouts built according to these schemes: analogous (**b**), monochromatic (**c**), triadic (**d**), and complementary (**e**).

Therefore, we developed 12 colour schemes for this website based on three primary colours: blue, green, and red (Figure 1, Figure 2 and Figure 3). Four types of colour schemes were developed for each primary colour: analogous, monochromatic, triadic, and complementary (Figure 1, Figure 2 and Figure 3).

### 2.3. Assessing the Contrast in Brightness Between Interface Elements

One of the key factors of visual accessibility is the contrast between interface elements, in particular, the brightness contrast index [12,13,14,15].

Therefore, in addition to the standard contrast check according to WCAG requirements, carried out at the stage of web page layout, this study conducted an additional analysis of the brightness contrast between key pairs of interface elements: text/background, buttons/background, and icons/background. Considering that in this design, the text or button is perceived as a separate figure (symbol) on the background, the calculation of the contrast was carried out according to the Weber formula [28]:

(12) $$C_{W}=\frac{L_{background}-\;L_{symbol}\;\;}{L_{background}},$$

To calculate the brightness of symbols ($L_{symbol}$) and background ($L_{background}$), the formulas for converting colors from RGB to CIELab model (4)–(9) were used.

For each colour scheme, a step-by-step calculation of contrast was performed using the Weber formula. Firstly, the contrast between the key elements reproduced in the colours of the corresponding scheme and the light background colour was calculated (colours vs. neutral background). The obtained values were averaged within each colour scheme (${\bar{C}}_{W}^{col-bg}$ value). Next, the inverse contrast between the light neutral background colour and the other colours of the scheme was determined (neutral background vs. colours) (${\bar{C}}_{W}^{bg-col}$ value). In addition, pairwise contrast calculations were performed between each colour and all other colours within each scheme (primary colour vs. all other colours, secondary colour vs. all other colours, tertiary colour vs. all other colours, neutral text colour vs. all other colours, and accent text colour vs. all other colours). All absolute values of pairwise contrast were averaged to calculate pairwise intra-scheme contrast ($\left|{\bar{C}}_{W}^{col-col}\right|$ value). The overall average absolute contrast value for each colour scheme was also determined, which was subsequently used for correlation analysis with the respondents’ ratings. To do this: within each colour scheme, the average contrast level ($\left|{\bar{C}}_{W}^{av}\right|$ value) was calculated by averaging all Weber contrast values of colour pairs in each scheme.

The calculations enabled us to determine the contrast of interface elements against the background, allowing for a quantitative assessment of the overall accessibility of a web page. It also enables us to test the hypothesis that the accessibility assessment is related to the level of contrast of elements on a webpage.

### 2.4. Preparation of Respondents

This study involved 18 men of different ages (from 17 to 58) with different types of colour blindness. They underwent appropriate tests to confirm the type and level of colour perception disorder (Ishihara test [29]). The study participants were divided into groups according to their type of colour blindness: deuteranopia (10 people), protanopia (5 people), and tritanopia (3 people).

### 2.5. Survey

The purpose of the survey was to evaluate the respondents’ assessment of webpages designed using different types of colour schemes (analogous, monochromatic, triadic, and complementary) with different primary colours according to the following criteria:

- Clarity of information perception (on a scale from 1 to 5, where 1 indicates difficulty in perceiving information, and 5 indicates ease in perceiving information);
- Visibility of essential page elements (navigation components and buttons) (on a scale from 1 to 5, where 1—key elements cannot be distinguished from others, and 5—key elements are clearly distinguished from others);
- Aesthetic appeal (on a scale from 1 to 5, where 1 is aesthetically disappointed, and 5 is aesthetically satisfied).

A survey was conducted to assess the importance of various criteria from the perspectives of both leading industry experts—specifically, UX/UI designers involved in inclusive design—and the survey respondents themselves. To acknowledge potential differences in opinions between these two groups regarding the significance of the criteria, a methodology was created to calculate an integrated weighted mean score for accessibility. This approach incorporates double weighting to ensure a comprehensive evaluation [30].

### 2.6. Data Analysis

The proposed method for calculating the integrated weighted mean score for accessibility, denoted as ${\bar{X}}_{ijkl}$, is based on the scores $x_{ijkl}$ provided by each *i*-th respondent (*i* ∈ {1, 2, …, 18}). This scoring system considers the *j*-th type of colour vision impairment (*j* ∈ {1, 2, 3}), where 1 represents deuteranopia/deuteranomaly, 2 signifies protanopia/protanomaly, and 3 indicates tritanopia/tritanomaly. The analysis encompasses four different colour schemes (*k* ∈ {1, 2, 3, 4}), 1 for analogous, 2 for monochromatic, 3 for triadic, and 4 for complementary colour schemes, which are based on a primary colour (*l* ∈ {1, 2, 3}): 1 for red, 2 for green, and 3 for blue. Furthermore, the assessment relies on three criteria (*c* ∈ {1, 2, 3}): 1 for clarity of information perception, 2 for visibility of essential page elements, and 3 for aesthetic appeal.

The weight coefficients from respondents $w_{c}^{\left(r\right)}$ and experts $w_{c}^{\left(e\right)}$ are normalised and combined to determine the weighted mean score of each colour scheme’s accessibility. The sum normalisation method was used to normalise the weights as follows:

(13) $${\tilde{w}}_{c}^{\left(r\right)}=\frac{w_{c}^{\left(r\right)}}{\sum_{c=1}^{3}w_{c}^{\left(r\right)}},\;{\tilde{w}}_{c}^{\left(e\right)}=\frac{w_{c}^{\left(e\right)}}{\sum_{c=1}^{3}w_{c}^{\left(e\right)}}$$

The analysis considers the varying importance of opinions from experts and respondents regarding the significance of criteria for evaluating colour schemes. It recognises that respondents have different types of colour vision deviations and personal experiences of colour perception, which cannot be fully understood by experts or replaced by their theoretical knowledge. Additionally, the survey involved a larger number of respondents than experts, further enhancing the representativeness and reliability of the feedback on criterion weights from respondents. While the opinions of experts are crucial for technical analysis and provide a professional perspective, the following weighting coefficients for respondents, *α*, and for experts, *β*, are proposed:

(14) α=0.7,β=0.3, (α>β, α+β=1),

The next step was to determine the combined weight for each indicator and normalise it:

(15) $$w_{c}^{int}=\alpha \cdot {\tilde{w}}_{c}^{\left(r\right)}+\beta \cdot {\tilde{w}}_{c}^{\left(e\right)},\;{\tilde{w}}_{c}=\frac{w_{c}^{int}}{\sum_{c=1}^{3}w_{c}^{int}}$$

Thus, based on the obtained estimates $x_{ijkl}$ and considering the defined integrated weighting coefficients ${\tilde{w}}_{c}$, the integrated weighted mean score for accessibility ${\bar{X}}_{ijkl}$ was calculated as follows:

(16) $${\bar{X}}_{ijkl}=\sum_{c=1}^{3}{\tilde{w}}_{c}\cdot x_{ijkl}$$

For the next step, a statistical analysis was performed based on the weighted average scores of respondents with different types of colour vision disorders (deuteranopia/deuteranomaly, protanopia/protanomaly, and tritanopia/tritanomaly) for each colour scheme (analogous, monochromatic, triadic, and complementary), which were also constructed using different colours (red, green, and blue). Specifically, a two-way repeated-measure ANOVA was conducted [31]. This analysis was carried out twice.

Initially, the importance of factors such as the type of colour scheme and the primary colour was determined when assessing the accessibility of webpages for people with a certain type of colour blindness. Therefore, the model structure for this analysis was as follows:

(17) $${\bar{X}}_{ijkl}=\mu +\alpha_{k}+\beta_{l}+{\left(\alpha \beta \right)}_{kl}+\varepsilon_{ijkl},$$

where *μ* is the average score in the entire sample, $\alpha_{k}$ is the effect of the type of colour scheme, $\beta_{l}$ is the effect of the primary colour, ${\left(\alpha \beta \right)}_{kl}$ is the effect of the interaction of factors, and $\varepsilon_{ijkl}$ is the residual error.

The next stage was the determination of the influence of the type of colour scheme based on a specific primary colour and the type of colour vision deviation on the weighted mean score for the accessibility of webpages. With this analysis, the model structure looks as follows:

(18) $${\bar{X}}_{ijkl}=\mu +\gamma_{kl}+\delta_{j}+{\left(\gamma \delta \right)}_{kl,j}+\varepsilon_{ijkl},$$

where $\gamma_{kl}$ is the effect of the type of colour scheme based on a specific primary colour, $\delta_{j}$ is the effect of the type of colour vision deviation, ${\left(\gamma \delta \right)}_{kl,j}$ is the effect of the interaction of factors, and $\varepsilon_{ijkl}$ is the residual error. The purpose of this analysis was to identify how the perception of respondents with different types of colour vision deviation changes for each colour scheme, taking the primary colour into account.

### 2.7. Limitations of This Study

This study has certain limitations. First, the sample size was relatively small, with only 18 people involved in this study. This sample size can affect the statistical significance of the results, and it is highly dependent on the random personal colour perception of an individual respondent. To improve the accuracy and reliability of the results, a larger sample of respondents should be involved in the future.

Second, this study involved exclusively male adult audiences. That may limit the applicability of the findings to women and children. Their preferences and perceptions of colour schemes may differ, so involving children and women in similar studies in the future could provide a more balanced and comprehensive understanding of the topic.

Thirdly, this study focuses on a single page of a website, which limits its applicability to assessing the overall design quality of an entire website. The design of other pages may differ significantly, with different functional elements, structures, and use of colour schemes. This can affect user perception. Future research should consider several pages with different functionalities to evaluate how colour schemes work in different contexts.

Additionally, due to the online format of the survey, it was not possible to conduct individual analyses of contrast sensitivity according to Weber’s law within the framework of this study. Such an analysis requires conducting specialised offline visual tests for each respondent. Since these factors cannot be standardised remotely, the use of Weber’s law as a tool for individual assessment remained outside the scope of the chosen methodology. Instead, objective calculations of the contrast between the colours of the colour schemes were used for quantitative assessment and correlated with a subjective visual assessment of the accessibility of a web page built using the corresponding colour scheme.

These limitations should be taken into account when planning future experiments to obtain more representative and comprehensive data.

## 3. Results

### 3.1. The Results of the Study of the Level of Distortion of Different Colours in Perception by People with Different Types of Colour Blindness

A survey of respondents on the accuracy of colour reproduction in colour blindness simulators showed that Sim Daltonism and Coblis Colour Blindness Simulator provide the most reliable simulations. Although the results of the simulation in Colour Oracle are generally similar to those of the above programs, respondents noted that this tool slightly reduces colour saturation for them, making them look more “pale.” Therefore, the results of computer colour simulation using the Coblis Colour Blindness Simulator tool were used for further analysis. It was used to obtain a set of colours modelled according to different types of colour perception disorders.

A total of 128 colours were analysed using the simulator. Table 1 shows three typical examples from each colour group, simulated according to the type of colour perception disorder. The table visually represents the colours and their simulations with their corresponding hexadecimal codes. This helps to visualise the level of perceptual distortion of each colour.

**Figure 4:**
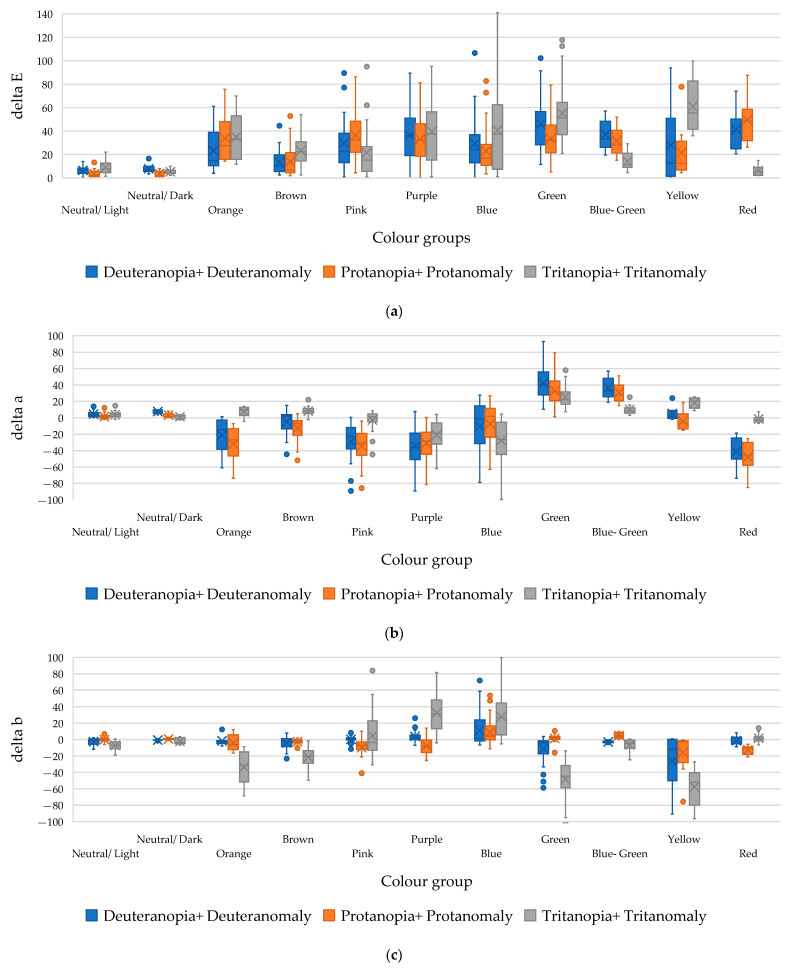
The calculated average values of deviation in the perception of different colour groups by people with various types of colour blindness: (**a**) colour deviation ΔE, (**b**) the red/green coordinate deviation Δa, and (**c**) the yellow/blue coordinate deviation Δb.

To objectify the assessment of the level of distortion of perception of individual groups of colours, the colour deviation of the colour and its components were calculated according to the proposed method. The graphs in Figure 4 objectively demonstrate which colour spectrum is distorted in the perception of people with different types of colour vision disorders, the extent of the distortion, and how it occurs.

The calculated colour deviation values for each colour group were averaged within the boundaries and are presented in Figure 4a. The graph shows that red and its derivatives (violet and pink), as well as the green and cyan colour groups, undergo significant distortions in perception in protanopia, protanomaly, deuteranopia, and deuteranomaly. In tritanopia and tritanomaly, yellow and blue colours are most distorted, resulting in significant distortion of green and purple colour groups.

For each colour, the value of the lightness coordinates *L*, *a*, and *b* in the CIELab space was determined for both normal vision and for models with colour perception disorders (protanopia and protanomaly, deuteranopia and deuteranomaly, and tritanopia and tritanomaly), simulated using the CVD simulation generator. The analysis showed that the changes in the *L* coordinate between the conditionally standard and simulated forms of vision were insignificant, i.e., colour perception disorders practically do not affect the perception of colour lightness. This result indicates that the *L* component is a relatively stable indicator, regardless of the type of colour blindness, and the main distortions of perception relate to the coordinates *a* and *b*, which correspond to the colour tone.

Figure 4b,c demonstrate which component of colour has the most significant impact on its distortion when perceived by people with different types of colour blindness and how this occurs.

An increase in the *a* index (when *a* > 0) indicates a shift in the perception of the group of corresponding colours to the red spectrum, while a decrease in the *a* index (when *a* < 0) indicates a shift in the group of colours to the green spectrum.

An increase in the b index (when *b* > 0) indicates a shift in the perception of the corresponding group of colours to the yellow spectrum, and a decrease in the *b* index (when *b* < 0) indicates a shift in the perception of colours to the blue spectrum.

**Figure 5:**
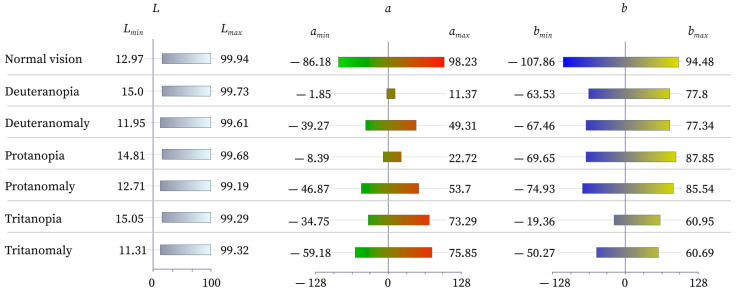
The ranges of coordinates *L*, *a*, and *b* for the studied colours in the perception of individuals with normal vision and individuals with various types of colour vision deviations.

Figure 5 shows the ranges of coordinates *L*, *a*, and *b* of the CIELab colour space for observers with normal vision and with various colour vision deviations. The graph is built based on the studied colours, and it allows for visual assessment of which colour vision deviation—which colour spectrum—undergoes the most significant distortion. It is possible to determine the limits of each coordinate, which will indicate the maximum capabilities of people with colour vision deviations to perceive the corresponding colour spectrum. In particular, it is seen that in deuteranopia and protanopia, there is a narrowing in the range of the coordinate a, which indicates a decrease in the perception of red–green shades. For tritanopia, the limitation is mainly manifested in the range of the coordinate *b*, which is responsible for the blue–yellow axis. This visual analysis allows for the tracing of how the colour coverage changes for each type of violation, and it is the basis for further correction of colour schemes in design.

### 3.2. The Results of a Survey of People with Different Types of Colour Blindness on Their Perception of Different Types of Colour Schemes

To identify significant factors influencing the weighted mean score for accessibility of different colour schemes applied to a specific webpage by individuals with various types of colour blindness, a comprehensive two-way repeated-measure ANOVA was employed. This thorough analysis enabled us to determine the impact of each factor and its interaction on the weighted mean score for the accessibility of webpages built using different colour schemes based on various primary colours.

In the first stage, a two-factor analysis of variance was conducted to assess the impact of two independent variables—primary colour (red, green, and blue) and type of colour scheme (analogous, monochromatic, triadic, and complementary)—on the weighted mean score of webpage accessibility. The impacts of the factors and their interactions were analysed separately for each type of colour deviation.

The analysis revealed that the considered factors and their interactions are significant (in all cases, *p*-*value* < 0.005, *F-value* > *critical F-value*) for different types of colour vision impairment (Table 2). This implies that the choice of primary colour in the colour scheme, the type of colour scheme, or the combination of primary colour and type of colour scheme can have a tangible impact on the accessibility of the webpage, thereby influencing user experience and inclusivity.

According to the analysis of variance (Table 2), it was determined that both factors—the primary colour and the type of colour scheme—have a significant impact on the weighted mean score of webpage accessibility for people with deuteranopia and deuteranomaly. At the same time, the primary colour of the scheme is a more significant factor of influence (*F-value* = 220.29 with *critical F-value* = 3.08, *p*-*value* = 7.68 × 10^−39^) than the type of colour scheme (*F-value* = 31.15, with *critical F-value* = 2.69, *p*-*value* = 1.37 × 10^−14^).

**Figure 6:**
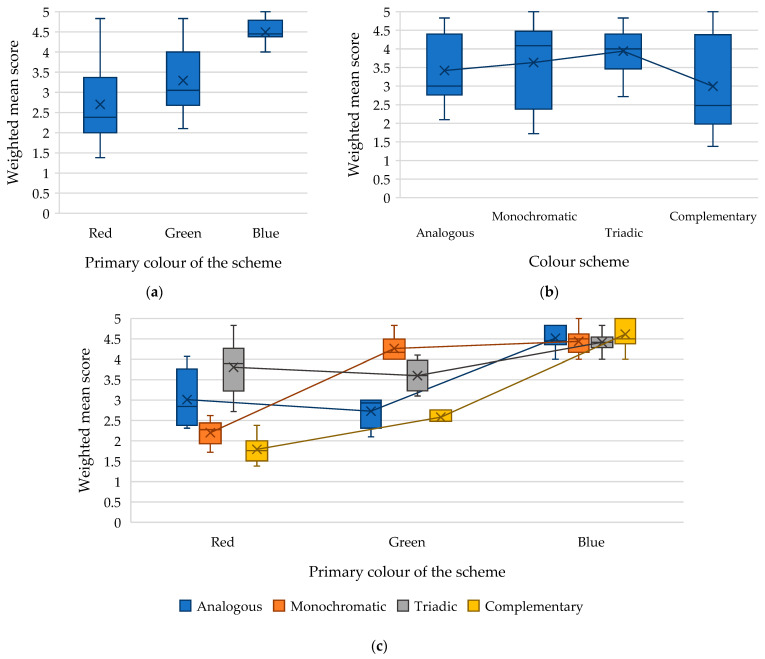
Dependence of the weighted mean score for the accessibility of a webpage by people with deuteranopia and deuteranomaly on the following: (**a**) the primary colour of the scheme; (**b**) the type of colour scheme; (**c**) the interaction of the factors “primary colour x colour scheme.”.

**Figure 7:**
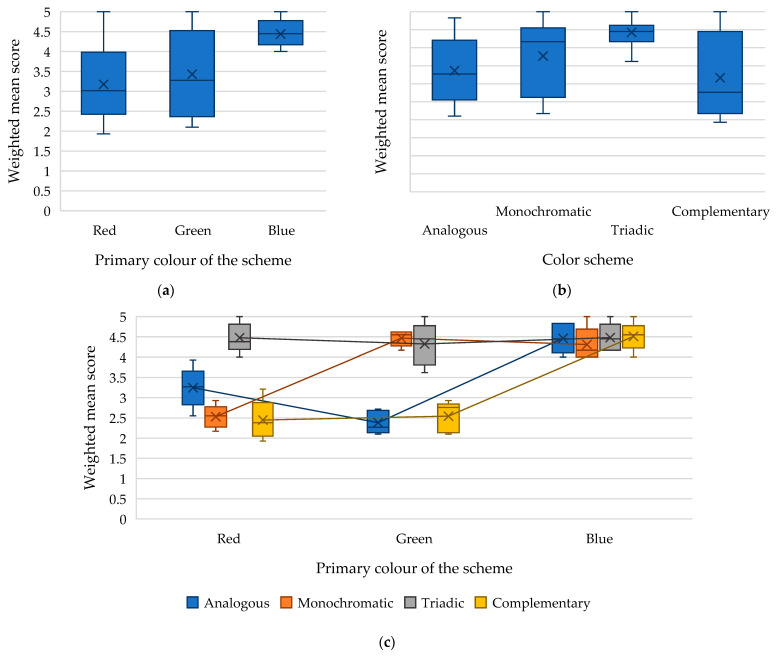
Dependence of the weighted mean score for the accessibility of a webpage by people with protanopia and protanomaly on the following: (**a**) the primary colour of the scheme; (**b**) the type of colour scheme; (**c**) the interaction of the factors “primary colour x colour scheme.”.

**Figure 8:**
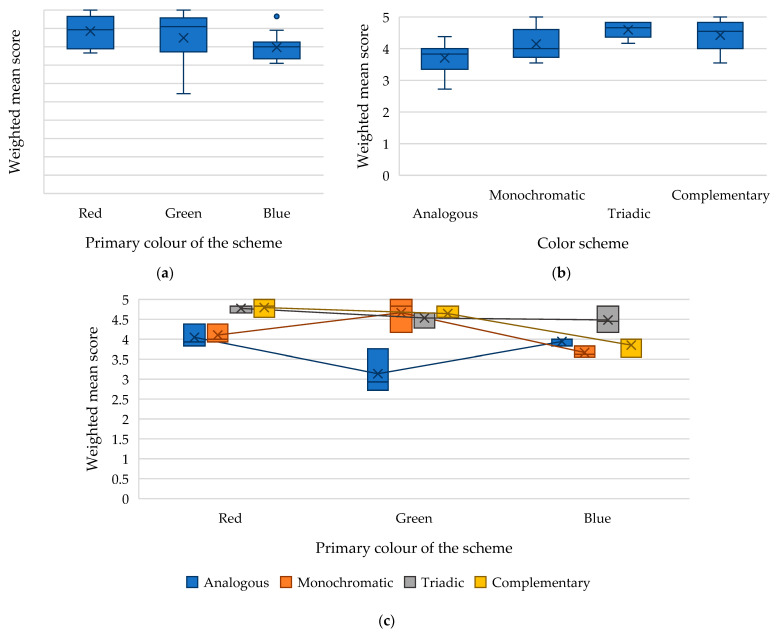
Dependence of the weighted mean score for the accessibility of a webpage by people with tritanopia and tritanomaly on the following: (**a**) the primary colour of the scheme; (**b**) the type of colour scheme; (**c**) the interaction of the factors “primary colour x colour scheme.”.

The results of the analysis are presented in Figure 6, Figure 7 and Figure 8, from which the impact of each factor and their interaction on the weighted mean accessibility score of a webpage built using different colour schemes based on various primary colours can be visually assessed.

Figure 6a shows that among individuals with deuteranopia and deuteranomaly, colour schemes with blue as the primary colour received the highest weighted mean score (average value: 4.51). Colour schemes with red as the primary colour were the least effective for individuals with deuteranopia and deuteranomaly (average value: 2.7).

The analysis revealed that individuals with deuteranopia and deuteranomaly tend to prefer triadic colour schemes, as indicated by the highest weighted mean score (average value: 3.94) (Figure 6b). The complementary colour scheme has the lowest weighted mean score (average value: 2.99).

There is a dependence of the assessments of respondents with deuteranopia and deuteranomaly on the interaction of the factors “primary colour × type of colour scheme” (*F-value* = 31.43 with *critical F-value* = 2.18, *p*-*value* = 1.29 × 10^−21^).

Figure 6c shows that when choosing red as the primary colour, it is preferable to opt for a triadic colour scheme (average score: 3.80) rather than a complementary colour scheme (average score: 1.79). When developing a design with a primary green colour, it is worth choosing a monochromatic colour scheme (average score: 4.26) and avoiding a complementary one (average score: 2.58). The low scores for complementary colour schemes that use red or green as the primary colour can be explained by the fact that this colour scheme includes colours that are opposite on the colour wheel (such as red and green). It can lead to confusion and difficulty in identifying and recognising corresponding elements for individuals with deuteranopia and deuteranomaly.

The monochromatic colour scheme with primary red (average value: 2.19) and the analogous colour scheme with primary green (average value: 2.72) also received relatively poor ratings, demonstrating the difficulty of distinguishing colours with low colour contrast.

Thus, the highest accessibility scores for people with deuteranopia and deuteranomaly were demonstrated by triadic colour schemes with a primary red colour (average value: 3.8) and a monochromatic colour scheme with primary green colour (average value: 4.26), as well as all types of studied colour schemes with a primary blue colour (the weighted average score of all schemes was over 4.3 points).

The high scores for the blue primary colour schemes are logical, as people with deuteranopia and deuteranomaly have no problem perceiving blue shades. However, it is worth noting that the triadic blue primary colour scheme has a relatively low score among the four colour schemes studied, which can be explained by the use of green and red in the design implied by this colour scheme.

The results of the analysis of variance of the assessment of the accessibility of webpages by people with protanopia and protanomaly showed that both factors—the primary colour and the type of colour scheme—as well as the interaction of these factors are also statistically significant. In particular, it was determined that the primary colour of the scheme has the greatest influence (*F-value* = 59.83 with *critical F-value* = 3.19, *p*-*value* = 9.19 × 10^−14^) in forming the weighted average assessment of the perception of webpages by people with protanopia and protanomaly (Table 2 and Figure 7).

Figure 7a shows that the highest scores were obtained by webpages built using colour schemes of various types with a primary colour of blue (average value: 4.44). In contrast, colour schemes with a primary colour of red received the lowest scores (average value: 3.17).

The influence of the type of colour scheme on the weighted mean score for the accessibility of webpages was found to be significant (*F-value* = 30.97, with a *critical F-value* of 2.80, *p*-*value* = 2.75 × 10^−11^). In particular, it was determined that people with protanopia and protanomaly perceive the triadic colour scheme best (average value: 4.43). In contrast, the complementary one is perceived as the worst (average value: 3.17) (Figure 7b). This suggests that individuals with these conditions perceive greater colour contrast in design as more accessible.

The interaction between the primary colour and the colour scheme also has a significant impact on the weighted mean score of webpage accessibility for individuals with protanopia and protanomaly (Figure 7c). Specifically, in cases where the primary colour of the webpage is red, the best option is a triadic colour scheme (average value: 4.47), and the worst option is a complementary one (average value: 2.44). For webpages with green as the primary colour, the recommended choices are either a monochromatic (average value: 4.46) or a triadic colour scheme (average value: 4.32). It is advisable to avoid analogous (average value: 2.38) and complementary colour schemes (average value: 2.54). In the case of using blue as the primary colour, any of the considered colour schemes is good, since all options achieved a mean score exceeding 4.3 points.

The low weighted average score for a monochromatic colour scheme with a primary red colour (average value: 2.53) indicates the difficulty in distinguishing shades of red for people with protanopia and protanomaly, which negatively affects the visibility of key elements and, therefore, the accessibility of the webpage. The low weighted mean score for the analogous colour scheme with the primary green colour (average value: 2.38) is also due to the appearance of non-contrast colours, which affected the low visibility of key elements on the page. People with protanopia and protanomaly find it challenging to interpret information on pages that use complementary colour schemes centred on red (average value: 2.44) and green (average value: 2.54). This issue can again be attributed to confusion between these colours, as such schemes often incorporate both colours simultaneously in their design.

Analysis of variance of the evaluation of webpages built using different colour schemes by people with tritanopia and tritanomaly demonstrated a significant effect of both the primary colour (*F-value* = 7.27 with *critical F-value* of 3.40, *p*-*value* = 0.003404) and the type of colour scheme (*F-value* = 16.57 with *critical F-value* of 3.01, *p*-*value* = 4.78 × 10^−6^), as well as their interaction (*F-value* = 7.11 with *critical F-value* of 2.51, *p*-*value* = 0.00019) on the weighted mean score for the accessibility of these webpages (Table 2, Figure 8).

Figure 8a shows that webpages built using colour schemes based on the red primary colour were the most accessible for people with tritanopia and tritanomaly (average value: 4.42). Meanwhile, webpages built using colour schemes based on the blue primary colour received the lowest scores (average value: 3.98).

Through an analysis of webpage ratings by individuals with tritanopia and tritanomaly, it was determined that this group found triadic colour schemes to be the most accessible (average score of 4.59) and analogous colour schemes to be the least accessible (average score of 3.7) (Figure 8b).

As noted above, analysis of variance also revealed a significant interaction effect between the factors “primary colour x colour scheme” on the weighted average assessment of webpage accessibility for people with tritanopia and tritanomaly. In particular, the analysis of the results (Figure 8c) revealed that this group views the analogous colour scheme with primary green the least favourably, receiving a weighted mean score of 3.13 points. This outcome can be linked to the inclusion of green, yellow, and blue in the scheme, which tends to confuse individuals with tritanopia and tritanomaly. Monochromatic and complementary colour schemes featuring primary blue also received low ratings (3.66 and 3.85, respectively), highlighting the challenges faced by individuals with tritanopia and tritanomaly in distinguishing shades of blue and yellow.

Complementary colour schemes featuring primary red (average value of weighted mean score: 4.79) and green (average value of weighted mean score: 4.64) received the highest ratings, confirming that individuals with tritanopia and tritanomaly can easily perceive red and green colours.

**Figure 9:**
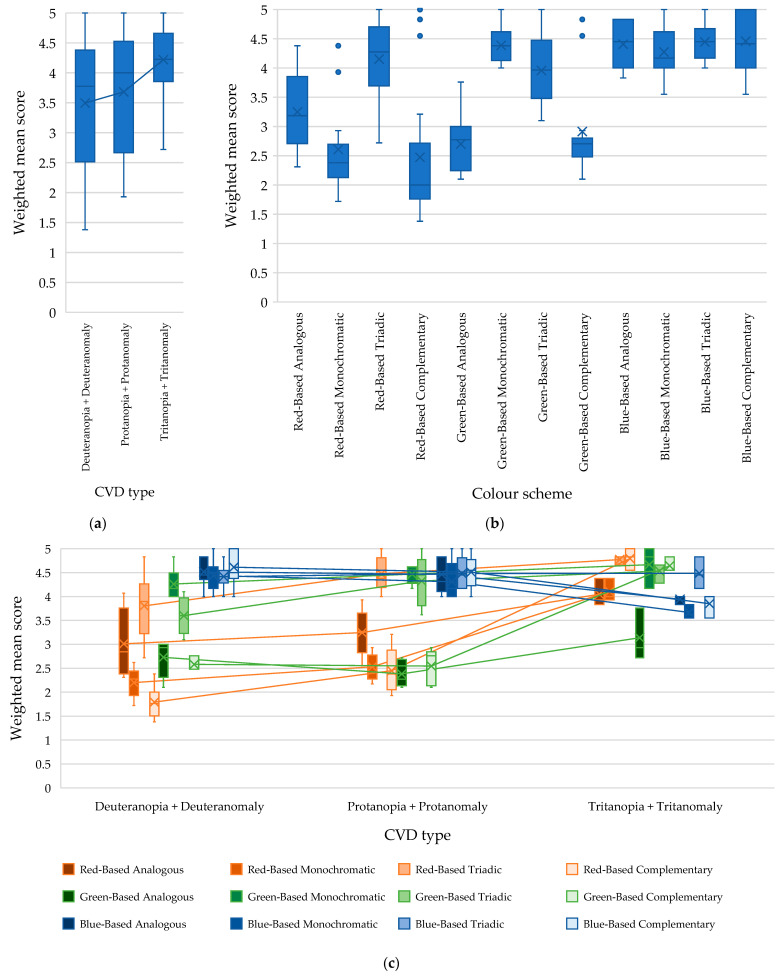
Dependence of the weighted mean score for the accessibility of a webpage by people with different types of CVD (colour vision deficiency) on the following: (**a**) the type of CVD; (**b**) the colour scheme; (**c**) the interaction of the factors “type of CVD x colour scheme.”.

At the second stage of this study, two-way repeated-measure ANOVA was conducted to identify the influence of the type of colour vision impairment (deuteranopia and deuteranomaly, protanopia and protanomaly, and tritanopia and tritanomaly) and the colour scheme with the primary colour (12 colour schemes in total) on the weighted mean score for the accessibility of webpages (Table 3 and Figure 9).

In Figure 9a, it can be seen that respondents with tritanopia and tritanomaly rated the accessibility of webpages built using different colour schemes higher (the average value of the weighted mean score is 4.22) than respondents with other types of colour vision deficiency.

As a result of this analysis, it was found that the type of colour vision deviation (*F-value* = 18.94 with *critical F-value* of 2.28, *p*-*value* = 1.91 × 10^−14^), the colour scheme (*F-value* = 74.85 with *critical F-value* of 1.86, *p*-*value* = 6.96 × 10^−54^), and their interaction (*F-value* = 4.77 with *critical F-value* of 1.42, *p*-*value* = 3.09 × 10^−14^) are statistically significant factors that form the weighted average assessment of webpage accessibility. The colour scheme has a greater impact on the weighted average assessment of webpage accessibility than the type of colour vision deviation.

Figure 9b illustrates the impact of the type of colour scheme based on a specific primary colour on the weighted mean score for the accessibility of a webpage according to all respondents, regardless of the type of colour vision impairment. This diagram enables us to identify general trends in the perception of colour design effectiveness among individuals with colour blindness, regardless of the specific type of deviation. Based on these results, web designers can make decisions about a universally designed website that is acceptable to most users.

Figure 9c presents a detailed visual assessment of the impact of combining scheme type and primary colour on the accessibility level of webpages for individuals with a specific type of colour vision impairment. Thus, the analysis enables us to identify not only general trends but also the different vector responses of individual user groups, which is very important from the perspective of developing personalised and accessible interfaces for people with colour vision impairments.

The analysis results indicate that triadic colour schemes are the most effective for all types of colour blindness. This underscores the importance of contrasting combinations that provide good visibility for individuals with colour blindness while also creating a harmonious visual effect.

Monochromatic schemes yield fairly good results, although they are less efficient than triadic schemes. Monochromatic colours, such as blues and greens, offer adequate contrast for users with colour blindness but do not provide the same level of element visibility or aesthetic satisfaction as triadic schemes.

Analogous colour schemes are generally less effective. That is especially true for red and green analogous colour palettes, where individuals with deuteranopia and protanopia find it challenging to distinguish colours, resulting in lower scores. These schemes perform poorly across all criteria, including the visibility of essential elements and overall aesthetic satisfaction. That suggests that these colour schemes are not optimal for individuals with red–green colour perception disorders.

Choosing complementary colour schemes requires caution, as their accessibility depends on the primary colour and the type of colour blindness. Complementary colour schemes featuring red and green as primary colours received the lowest ratings from individuals with deuteranopia and protanopia. In contrast, they received relatively high ratings from those with tritanopia and tritanomaly. Conversely, complementary colour schemes with a primary blue colour earned the lowest scores from individuals with tritanopia and significantly higher scores from people with deuteranopia and protanopia.

### 3.3. Results of Calculating the Contrast of Interface Elements

Contrast was measured according to Weber’s Formula (12) for each colour pair within each colour scheme. The results of the calculations are presented in Table 4. This enables the objective evaluation of the visual accessibility of content on a webpage.

The results of the calculations enabled us to quantitatively assess the compliance of the studied colour schemes with the visual accessibility criteria. In particular, the obtained average values of Weber contrast $({\bar{C}}_{W}\geq 0.5$) indicate that the majority of the used colour pairs belong to the categories of medium and high contrast. That, in turn, is an indicator of the potential convenience of perceiving interface elements by users, particularly those with colour vision impairment.

**Figure 10:**
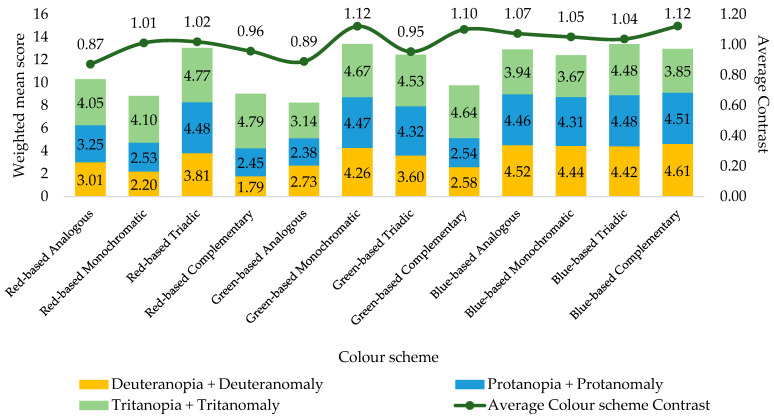
Correlation between the weighted average web page accessibility score obtained by individuals with different types of CVD and the overall average contrast of the colour scheme.

The quantitative contrast score was also used as an objective metric for correlation analysis with the subjective assessments of respondents. In particular, Figure 10 presents a diagram that demonstrates the correlation between the weighted mean score for the accessibility of a webpage by people with different types of CVD (colour vision deficiency) and the corresponding value of the overall average absolute contrast for each colour scheme.

Figure 10 shows a moderately positive correlation between the average contrast of a colour scheme and the weighted average accessibility ratings for individuals with different types of CVD. This indicates a general trend—with increasing contrast, the probability of perceiving a scheme as accessible increases. However, there are exceptions: red-based monochromatic and green-based complementary colour schemes received relatively low ratings from respondents but at the same time demonstrate high contrast values.

This confirms our hypothesis that the accessibility of web resources for individuals with different types of CVD depends not only on contrast but also on the type of colour combinations.

## 4. Discussion and Conclusions

The respondents with colour vision impairments supported the idea presented by the authors of [10] that colour in interface designs is crucial for ensuring high-quality usability. However, they also pointed out that identifying colours in interface objects can often negatively affect information perception, as errors in colour combinations can hinder understanding and navigation. It is important to note that the authors of [10] did not aim to take into account the specific needs of people with colour vision impairments in their conclusions. Nevertheless, given the significant number of people worldwide who face such challenges, it is important to create interfaces that comply with the principles of universal design.

The needs of people with colour blindness have been highlighted by many researchers [11,12,13,14,15]. Still, in most cases, they recommend using a high-contrast mode of information reproduction that is oriented towards the WCAG recommendations. According to the above studies, this effectively ensures accessibility for people with colour vision impairments. However, based on the feedback of web designers and developers (also mentioned, for example, in [4]), it is often the case that the concept of a web resource or the designer’s idea does not utilise increased contrast of elements. Excessive contrast may contradict the interface’s general style and aesthetics, reducing the design’s visual appeal and harmony. In such situations, the authors of [15,16] recommend using additional means of visual identification, such as different textures, contours, pictograms, and pip drawings. However, again, this may violate the original design idea or overload the interface design.

Therefore, this study presents an investigation aimed at resolving the dilemma between ensuring accessibility and preserving the aesthetic integrity of the interface. In particular, a study was conducted to determine the optimal colour scheme to improve the accessibility of interfaces for people with different types of colour blindness. We also analysed the impact of the primary colour in all types of colour schemes on the accessibility of web resources for people with different types of colour blindness.

To further analyse the accessibility of colour schemes for users with colour vision impairments, the Weber average contrast score was added to the standard WCAG criteria. This enabled a quantitative comparison of colour schemes with varying degrees of accessibility. Further correlation between the average contrast and the weighted average accessibility score for different types of CVD showed a moderately positive relationship. This result suggests that higher contrast generally improves the accessibility of colour schemes for people with colour vision impairments. However, it was found that contrast is not the only determining factor—the type of colour scheme and the base colour also significantly affect the accessibility score.

The results can serve as a helpful guide for designers when choosing a colour palette, especially if their task is to adapt the design to meet the needs of a particular group of users with colour vision impairments.

In cases where the interface design focuses on specific types of colour blindness, it is advisable to consider the nuances of colour perception among users with different colour vision disorders. Accordingly, the following recommendations can be made.

For individuals with deuteranopia and deuteranomaly (green colour perception disorders), it is beneficial to choose a triadic colour scheme when red is the primary colour. This approach utilises three equidistant colours on the colour wheel, creating a balanced contrast that aids colour discrimination, even for those with difficulties perceiving the green spectrum. When green is the primary colour of the interface, employing a monochromatic colour scheme is recommended, which involves using various shades and saturations of the same colour to prevent confusion from mixing similar hues. If blue is the primary colour in the interface design, designers can freely select any colour scheme.

For individuals with protanopia and protanomaly (red colour perception disorders), it is advisable to use a triadic colour scheme that ensures sufficient contrast between interface elements if the primary colour of the interface is red. If the primary colour is green, it is recommended to select either a triadic or monochromatic scheme. Both options provide visual consistency and help prevent confusion between colours. If blue is the primary colour, designers can choose from a full range of colour schemes, as blue is generally well perceived by those with protanopia and protanomaly.

For people with tritanopia and tritanomaly, it is best to choose a complementary or triadic colour scheme if the primary colour is red. This will provide high contrast between interface elements and improve their recognition, even if the user’s perception of blue is limited. If the primary colour is green, it is worth choosing either a complementary or monochromatic colour scheme. This will ensure the design’s consistency and a high level of accessibility for people with tritanopia and tritanomaly. Designers should be careful when using blue as the primary colour, since the perception of blue itself is impaired in people with tritanopia and tritanomaly. The analysis highlights blue triadic schemes, which have shown excellent results for individuals with tritanopia. These remain among the most effective for this group, offering good contrast and ease of perception.

In general, we can conclude that triadic colour schemes are the most versatile and practical option for all types of colour blindness. They ensure the high visibility of elements, the easy perception of information, and the creation of aesthetically pleasing colour combinations. Monochromatic schemes can also be a good option, but they are inferior to triadic schemes in terms of efficiency. Complementary colour schemes require the careful selection of primary colours, as their perception depends on the type of colour blindness of the user. Analogous colour schemes should be used cautiously, as they are ineffective for people with protanopia and deuteranopia.

In this study, three primary colours—red, green, and blue—were selected as the main colours for the development of colour schemes that were subject to investigation. Therefore, the direction of further research is to use other colours as the primary ones and increase the number of options for colour scheme types. It is also essential to research the availability of interfaces that combine colour with additional elements for visual identification components.

## Figures and Tables

**Table 1:** Typical examples of colours simulated with the Coblis Colour Blindness Simulator for each colour group and according to the type of colour perception disorder.

Colour Groups	Mnemonic Colour Names	Colours for Normal Vision	Simulated Colours Based on the Type of Colour Perception DISORDER					
			Deuteranopia	Deuteranomaly	Protanopia	Prot-Anomaly	Tritanopia	Trit-Anomaly
Neutral/Dark	light grey	#d3d3d3	#e7ccd4	#dfcfd4	#d4d0d0	#d4d1d2	#d4d0e0	#d2d0da
	dark grey	#a9a9a9	#b9a4aa	#b3a6aa	#a8a4a4	#aaa7a8	#a9a6b3	#a6a4ac
	grey	#808080	#8c7c81	#887d81	#807d7d	#828180	#828089	#7f7d83
Neutral/Light	ivory	#fffff0	#fffefe	#fffef9	#fffefc	#eeeee8	#e0e4e6	#f3f4f0
	cornsilk	#fff8dc	#fff6f3	#fff7eb	#fef7e6	#fff6e3	#fff4ff	#fff5f3
	papaya whip	#ffefd5	#ffede7	#fde9de	#fbefd6	#fcf0d5	#ffebf9	#ffedec
Orange	orange	#ffa500	#eaaf00	#f0ab05	#d1b91a	#e0b010	#ff9ea8	#ff9f6c
	dark orange	#ff8c00	#daa300	#e29100	#baa511	#d29509	#ff7b84	#ff7a4e
	orange red	#ff4500	#b38700	#d37f1d	#aa9a36	#c68029	#fe575a	#ff422a
Brown	Navajo white	#ffdead	#ffdcc0	#feddb9	#efdfac	#f3ddaa	#fed7e4	#fcd6cd
	sienna	#a0522d	#826329	#996423	#756933	#856030	#a84e53	#a44c42
	brown	#a52a2a	#705522	#854c27	#625a39	#7a4a34	#a32f31	#a52e2f
Pink	pink	#ffc0cb	#e8caca	#f1c9cc	#d5d1d3	#e5cdd2	#ffc1cf	#ffc0cd
	light pink	#ffb6c1	#e1c3bf	#e9babf	#c5c3ce	#dcbfc9	#feaebc	#feaabb
	deep pink	#ff1493	#938288	#b75e98	#6985da	#a15ec3	#f84244	#fa2f6a
Purple	violet	#ee82ee	#99a8e7	#b99ae9	#8aa7fe	#b099f7	#e4919c	#ea82b8
	fuchsia	#ff00ff	#5793f0	#9b68f3	#6492ff	#9b65fe	#ec6b72	#f14ba3
	purple	#800080	#2c4a78	#4f327d	#074c97	#322f8f	#7e363a	#862957
Blue	light blue	#add8e6	#d9cbe9	#c6cfe8	#d0cfe0	#c2d2e2	#abd8e9	#abd8e8
	blue	#0000ff	#005288	#0337b0	#0351ac	#0938c3	#0a595c	#144296
	dark blue	#00008b	#002b48	#001d64	#002e60	#001c72	#003133	#00215b
Green	spring green	#00ff7f	#ffd4a4	#a1e398	#f2db76	#9de87d	#77eeff	#55f3d2
	green	#008000	#88671d	#526c10	#776900	#507306	#34747d	#227851
	olive	#808000	#ab8227	#9e8517	#98870d	#948b14	#92838c	#928661
Cyan (blue-green)	light cyan	#e0ffff	#fff6fc	#f4f9fd	#efeaed	#e0f6f9	#e0f7fd	#d1f9fd
	cyan	#00ffff	#e8dfff	#95edff	#e7e4f0	#93eff6	#9ff4ff	#66f9ff
	dark turquoise	#00ced1	#bbb4d7	#83c2d1	#c0bfc7	#88c7c1	#41d2e1	#16cdd8
Yellow	yellow	#ffff00	#f5ebe6	#f3f298	#faf1cc	#f9f47a	#f7eef4	#faf19d
	gold	#ffd700	#ffd38e	#ffd75d	#f8df27	#f5d60f	#fec8d3	#fbc987
	dark golden rod	#b8860b	#b58708	#bb890b	#9f8d11	#ac870e	#c77177	#c86d4a
Red	red	#ff0000	#a27a00	#c15702	#8f812c	#b5591c	#f42512	#f41e12
	crimson	#dc143c	#8c6b31	#a9533f	#766f55	#9a524e	#d62321	#d51e28
	maroon	#800000	#513d00	#642700	#494111	#5c290a	#7f0c00	#831009

**Table 2:** Results of the first two-factor analysis of variance (ANOVA) of webpage accessibility ratings from people with different types of colour vision impairment (factors: colour scheme type and primary colour).

CVD Type	Source of Variation	SS	df	MS	F-Value	*p*-Value	Critical F-Value
Deuteranopia + Deuteranomaly	Primary colour	66.88	2	33.44	220.29	7.68 × 10^−39^	3.08
	Colour scheme	14.19	3	4.73	31.15	1.37 × 10^−14^	2.69
	Interaction (colour × scheme)	28.63	6	4.77	31.43	1.29 × 10^−21^	2.18
	Residual (within groups)	16.39	108	0.15			
Protanopia + Protanomaly	Primary colour	17.95	2	8.98	59.83	9.19 × 10^−14^	3.19
	Colour scheme	13.94	3	4.65	30.97	2.75 × 10^−11^	2.80
	Interaction (colour × scheme)	18.19	6	3.03	20.21	1.25 × 10^−11^	2.29
	Residual (within groups)	7.20	48	0.15			
Tritanopia + Tritanomaly	Primary colour	1.19	2	0.60	7.27	0.003404	3.40
	Colour scheme	4.07	3	1.36	16.57	4.78 × 10^−06^	3.01
	Interaction (colour × scheme)	3.49	6	0.58	7.11	0.00019	2.51
	Residual (within groups)	1.97	24	0.08			

**Table 3:** Results of the second two-factor analysis of variance (ANOVA) of webpage accessibility ratings from people with different types of colour vision impairment (factors: type of colour vision deviation and colour scheme).

Source of Variation	SS	df	MS	F-Value	*p*-Value	Critical F-Value
CVD type	14.92	5.00	2.98	18.94	1.91 × 10^−14^	2.28
Colour scheme	129.69	11.00	11.79	74.85	6.96 × 10^−54^	1.86
Interaction (CVD type × scheme)	41.32	55.00	0.75	4.77	3.09 × 10^−14^	1.42
Residual (within groups)	22.68	144.00	0.16			

**Table 4:** Results of calculating the contrast of interface elements built using different colour schemes.

Primary Colour	Colour Scheme	Mean Weber Contrast (${\bar{C}}_{W}$) for Colour Pairs			
		Colours vs. Neutral Background $\left({\bar{C}}_{W}^{col-bg}\;\mathrm{Value}\right)$	Neutral Background vs. Colours $\left({\bar{C}}_{W}^{bg-col}\;\mathrm{Value}\right)$	Pairwise Intra-Scheme Contrast $\left(\left|{\bar{C}}_{W}^{col-col}\right|\;\mathrm{Value}\right)$	Average Colour Scheme Contrast $\left(\left|{\bar{C}}_{W}^{av}\right|\;\mathrm{Value}\right)$
Blue	Analogous	0.61	−2.49	1.15	1.07
	Monochromatic	0.56	−2.18	1.13	1.05
	Triadic	0.57	−2.22	1.12	1.04
	Complementary	0.50	−2.04	1.23	1.12
Green	Analogous	0.64	−2.40	0.93	0.89
	Monochromatic	0.51	−2.10	1.22	1.12
	Triadic	0.67	−2.70	1.00	0.95
	Complementary	0.53	−2.16	1.19	1.10
Red	Analogous	0.59	−2.16	0.92	0.87
	Monochromatic	0.56	−2.13	1.09	1.01
	Triadic	0.57	−2.19	1.09	1.02
	Complementary	0.56	−2.08	1.02	0.96

## Data Availability

The results of the calculations and the respondents’ evaluations are presented in in a Google spreadsheet: https://www.figma.com/design/ktW0jlXCX2o4WUDMmVNDCh/Article1?node-id=0-1&p=f (accessed on 1 July 2025). The webpage mockups used in this study are available in Figma at the link: https://docs.google.com/spreadsheets/d/1-Lb_rlQHfT2sCk1OuKOXFf5HeE2rB3lr/edit?gid=182429728#gid=182429728 (accessed on 1 July 2025).

## Abbreviations

The following abbreviations are used in this manuscript:

CVD	Colour vision deficiency
